# Correlative multiphoton-STED microscopy of podocyte calcium levels and slit diaphragm ultrastructure in the renal glomerulus

**DOI:** 10.1038/s41598-024-63507-9

**Published:** 2024-06-06

**Authors:** Eva Wiesner, Julia Binz-Lotter, Agnes Hackl, David Unnersjö-Jess, Nelli Rutkowski, Thomas Benzing, Matthias J. Hackl

**Affiliations:** 1 Department II of Internal Medicine and Center for Molecular Medicine Cologne, University of Cologne, Faculty of Medicine and University Hospital Cologne, Cologne, Germany; 2 Cluster of Excellence Cellular Stress Responses in Aging-Associated Diseases (CECAD), University of Cologne, Faculty of Medicine and University Hospital Cologne, Cologne, Germany; 3 Department of Pediatrics, University of Cologne, Faculty of Medicine and University Hospital Cologne, Cologne, Germany

**Keywords:** Fluorescence imaging, Podocytes

## Abstract

In recent years functional multiphoton (MP) imaging of vital mouse tissues and stimulation emission depletion (STED) imaging of optically cleared tissues allowed new insights into kidney biology. Here, we present a novel workflow where MP imaging of calcium signals can be combined with super-resolved STED imaging for morphological analysis of the slit diaphragm (SD) within the same glomerulus. Mice expressing the calcium indicator GCaMP3 in podocytes served as healthy controls or were challenged with two different doses of nephrotoxic serum (NTS). NTS induced glomerular damage in a dose dependent manner measured by shortening of SD length. In acute kidney slices (AKS) intracellular calcium levels increased upon disease but showed a high variation between glomeruli. We could not find a clear correlation between intracellular calcium levels and SD length in the same glomerulus. Remarkably, analysis of the SD morphology of glomeruli selected during MP calcium imaging revealed a higher percentage of completely disrupted SD architecture than estimated by STED imaging alone. Our novel co-imaging protocol is applicable to a broad range of research questions. It can be used with different tissues and is compatible with diverse reporters and target proteins.

## Introduction

Within the kidney, glomeruli are the functional filtration units consisting of different cell types such as mesangial cells, fenestrated endothelial cells, and podocytes. Podocytes are terminally differentiated epithelial cells lining the urinary site of the filtration barrier in the glomerulus. Podocytes consist of a large cell body with primary processes, from which smaller foot processes extend and enwrap the entire surface of glomerular capillaries. Neighboring foot processes interdigitate and are connected by a specialized cell-cell junction termed slit diaphragm (SD), containing unique proteins like nephrin and podocin^1,2^. The SD functions as a signaling hub and controls the podocyte’s complex cytoskeletal architecture^3^. Podocyte injury results in flattening and broadening of foot processes, called effacement, and podocyte detachment from the glomerular basement membrane. This is a major contributor to kidney disease development, progression and failure, as lost podocytes cannot be replaced, resulting in the clinical disease phenotype of focal segmental glomerulosclerosis. Human mutations in SD proteins, the actin-binding protein α-actinin-4, and the transient receptor cation channel 6 (TRPC6), regulating calcium signaling, result in the same phenotype of focal segmental glomerulosclerosis^4–6^. Besides TRP channels, podocytes are capable of purinergic calcium signaling whose activation results in increased intracellular calcium levels, underlining the importance of calcium, actin and the SD interplay for podocyte biology^7–10^. We have previously shown, using in vivo MP calcium imaging, that calcium levels in podocytes increase upon injury^11^. Similar results applying MP microscopy have been obtained ex vivo, using acute kidney slices (AKS), which provide a multicellular environment of vital intact kidney tissue^12^. The use of isolated glomeruli is limited for studying podocyte calcium levels as glomerular isolation induces high baseline calcium levels as a sign of podocyte injury^11^.

MP imaging uses a pulsed laser to excite fluorophores with multiple photons of a long wavelength. This allows for deeper tissue penetration especially as kidney tissue is highly scattering. However, as a light microscopy technique its resolution is limited by the diffraction limit of light. The gold standard for imaging of the SD has been electron microscopy, as the 30–40 nm width of the SD cannot be resolved by a conventional confocal microscope^13^. Super-resolution techniques like STED microscopy allow visualization and quantification of SD proteins after immunofluorescent labeling (Suppl. Fig S1)^14–16^. During image acquisition an additional donut-shaped stimulated emission depletion laser minimizes the area of fluorescence and thereby increases resolution^17^.

So far, functional calcium and structural SD data are obtained in separate experiments, prohibiting correlative studies in single glomeruli. In vivo STED imaging and MP coupled with STED (MP-STED) on a single microscope have been demonstrated but are not commercially available^18–21^. Additionally, in vivo STED imaging requires expression of fluorescent reporters and lacks the possibility of immunofluorescent staining. MP-STED imaging uses mostly fixed tissues, thus does not allow for imaging of live calcium dynamics.

To investigate how podocyte calcium levels and SD architecture are interlinked within one glomerulus in the transition from health to disease states, we established a new correlative imaging approach. It combines vital tissue and high-resolution imaging using MP and STED microscopy sequentially without the need for a combined MP-STED system. We applied our approach to compare podocyte calcium levels and SD architecture in control mice and mice treated with NTS. Injection of NTS leads to an autoimmune response against glomerular structures, resulting in podocyte injury.

## Results

**Figure 1:**
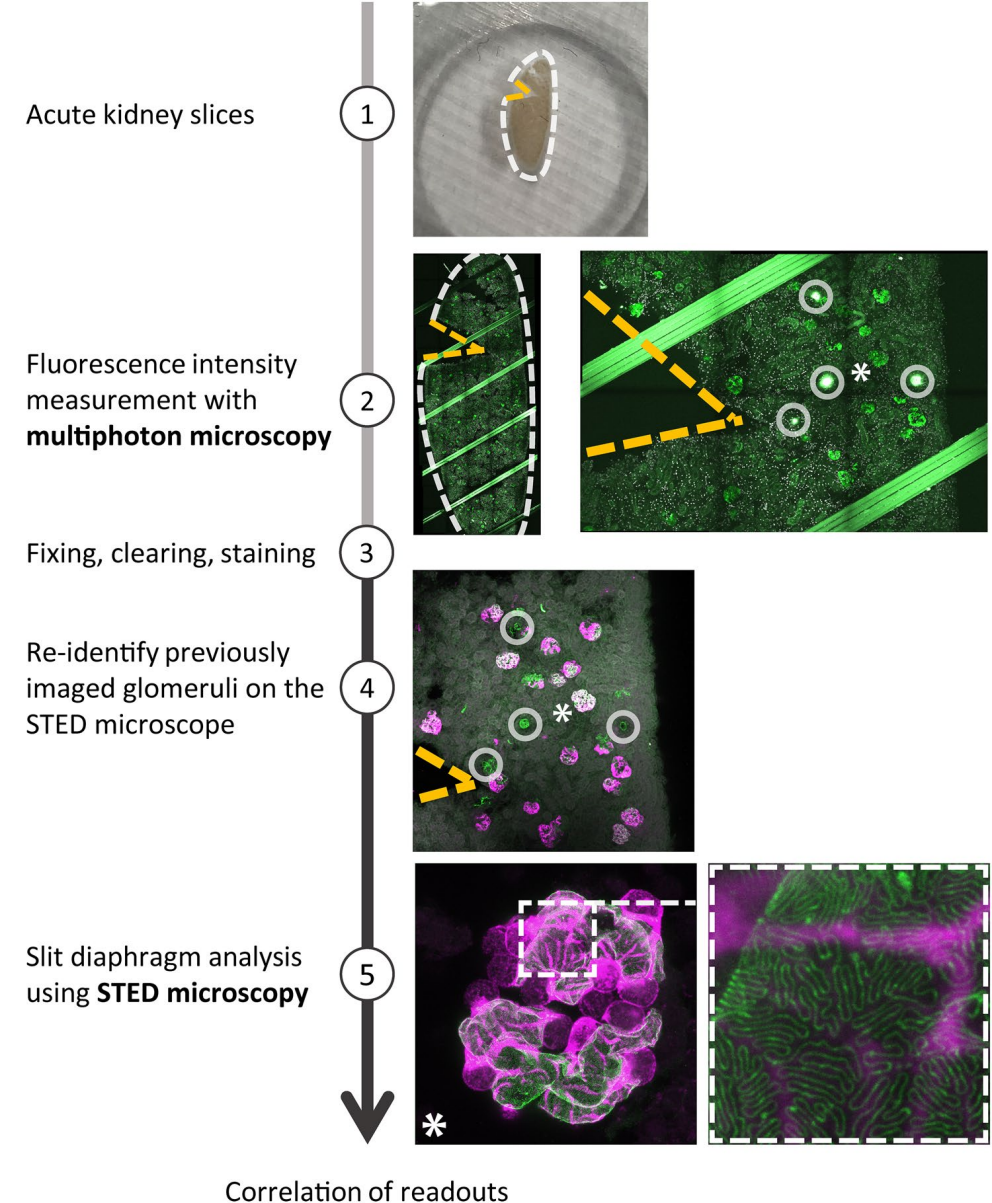
Workflow for correlative imaging of murine glomeruli using MP and STED microscopy. Overview of sample preparation and imaging steps. (**1**) AKS are marked by cutting a triangular shape at the upper left corner. (**2**) Intensity measurements with MP microscopy with burn marks as reference points (circles) and surrounding glomeruli (asterisk). The green channel shows endogenous GCaMP3 fluorescence and propidium iodide staining in white. (**3**) Fixation, clearing and staining of slices with goat anti-nephrin primary antibody, donkey anti-goat Alexa-594 secondary antibody and anti-GFP-Alexa647. (**4**) Confocal images with reference points (circles) and previously imaged glomeruli (asterisks). (**5**) STED images of the glomerulus marked with asterisk. Anti-GFP staining in magenta labelling GCaMP3 and anti-nephrin staining in green.

To combine MP and STED microscopy to measure podocyte calcium levels and SD morphology within the same glomerulus, we prepared AKS from mice expressing the calcium indicator GCaMP3 exclusively in podocytes^22,23^. The experimental imaging setup of our novel protocol is outlined in Fig. 1. A small mark in the AKS enabled identical slice orientation in both imaging modalities. During MP imaging, burn marks were positioned in safe proximity to glomeruli as reference points and large three-dimensional tile scans were acquired and served as an overview. In a single field of view z-stacks of single glomeruli were obtained to measure the mean GCaMP3 fluorescence, as a readout for calcium levels in podocytes. After MP imaging, AKS were fixed, the tissue was cleared and stained with antibodies against the SD protein nephrin and the green fluorescent protein (GFP), as the required clearing step resulted in complete loss of GCaMP3 fluorescence. The added reference points allowed the mapping of each glomerulus in the two data sets. Then, confocal image stacks were acquired to confirm morphology of previously imaged glomeruli. In the following image acquisition step, super resolution images of the nephrin pattern of those glomeruli were obtained with the activated STED laser. Finally, we quantified nephrin staining patterns as recently published by Butt et al. by measuring the SD length covering a capillary surface area^15^. This results in a numerical value for SD length/area, which has been shown to decrease in disease states in mice and humans compared to healthy controls.

**Figure 2:**
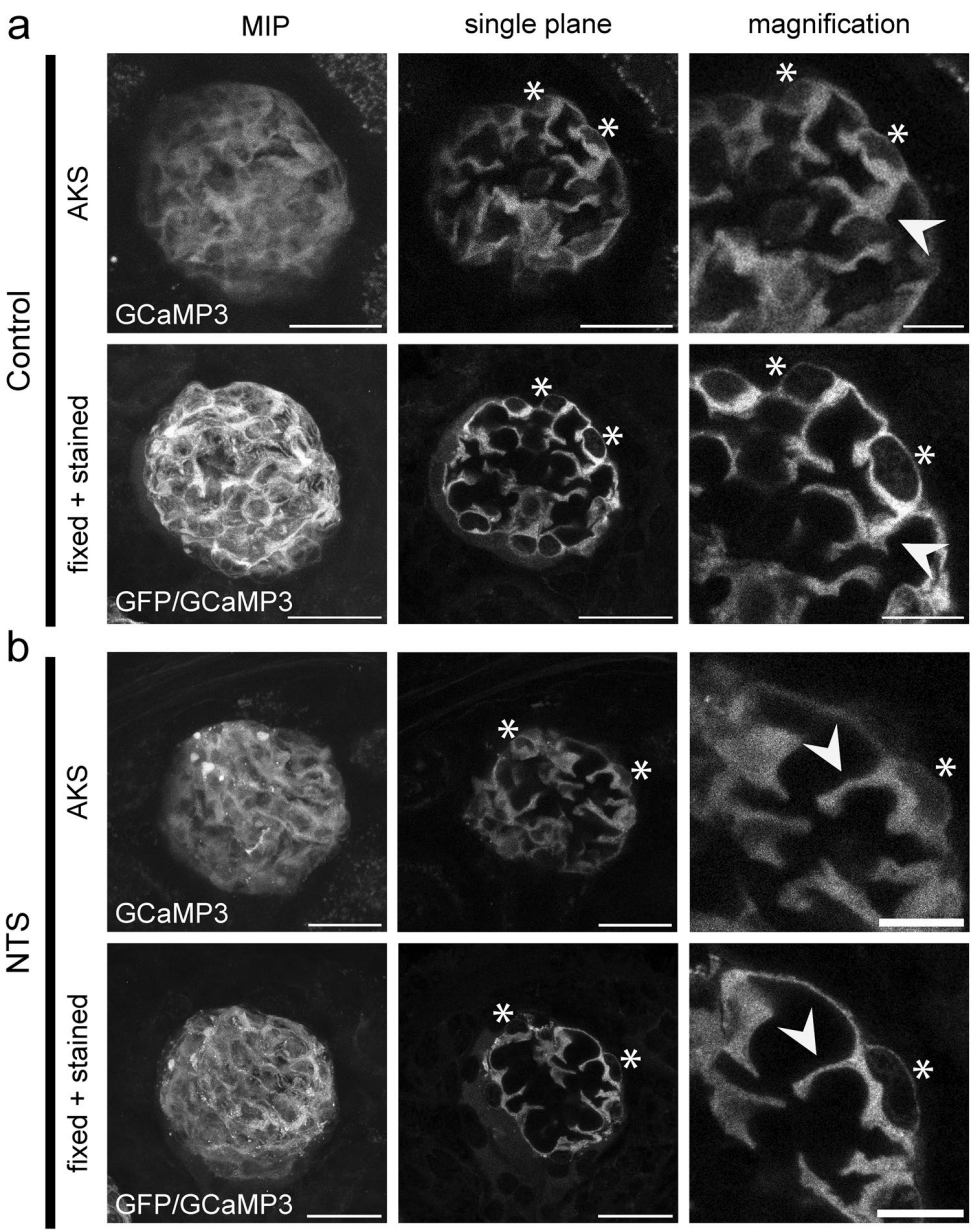
Reidentification of single mouse glomeruli and individual podocytes in AKS and after fixation, clearing and staining. Images of a control animal (**a**) and a NTS-treated mouse (11 µl/g body weight) (**b**) Images show the same glomerulus – from left to right – maximum intensity projection (MIP) of the whole glomerulus (left), single plane view (middle) and magnification of single podocytes (right). Endogenous GCaMP3 fluorescence for MP images (AKS) and anti-GFP/GCaMP3 signal for confocal images (fixed + stained). Arrowheads indicate podocyte primary processes. Asterisk indicating single podocyte cell bodies. Scale bar MIP & single plane – 25 µm; magnification – 10 µm.

Comparison of image stacks acquired by MP microscopy and stacks acquired with confocal settings on a STED microscope confirmed the overall conservation of tissue morphology (Fig. 2a MIP, Suppl. Video 1). We were able to match, both podocyte cell bodies (Fig. 2a, asterisks) and primary processes (Fig. 2a, arrows) in single planes in control animals in both imaging modalities and thus validated the possibility of studying the same glomerulus with both imaging modalities. We then applied our protocol to mice injected with a low dose (8 µl/g) or a high dose (11 µl/g) of NTS. Comparable to control tissue, we were able to identify corresponding podocyte cell bodies and processes in disease (Fig. 2b, shown for high dose NTS, 11 µl/g).

**Figure 3:**
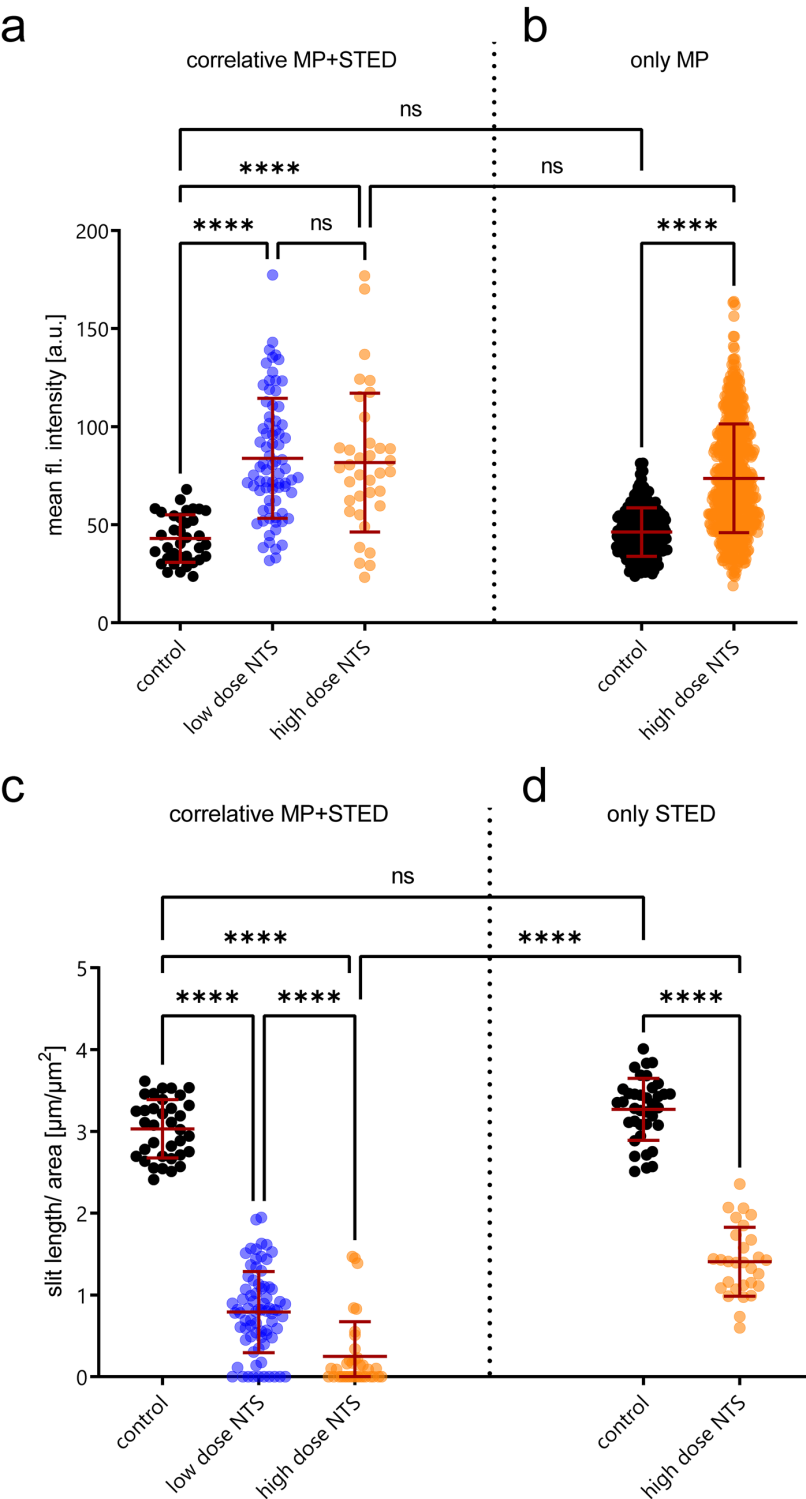
Correlative imaging reveals a stronger reduction of SD length/area than STED imaging alone. (**a**) Quantification of mean fluorescence (fl.) intensities of glomeruli for control and NTS-treated mice using our correlative MP + STED workflow. Control (n = 35 glomeruli of 3 mice), low dose NTS (8 µl/g, n = 76 glomeruli of 4 mice) and high dose NTS (11 µl/g, n = 36 glomeruli of 3 mice). (**b**) Quantification of mean fluorescence intensities of glomeruli for control and NTS-treated mice using only MP microscopy. Control (n = 228 glomeruli of 4 mice) and high dose NTS (11 µl/g, n = 578 of 5 mice). (**c**) Quantification of SD length/area of healthy control, low dose NTS (8 µl/g, n = 76 glomeruli of 4 mice) and high dose NTS (11 µl/g, n = 36 glomeruli of 3 mice). For each glomerulus, one STED image was acquired. Glomeruli in which no SD length could be measured were set to value = 0. (**d**) Quantification of SD length/area of healthy control and NTS-treated mice using STED microscopy only. Control (n = 37 glomeruli of 4 mice) and high dose NTS (11 µl/g, n = 29 glomeruli of 4 mice). Statistical analysis: ordinary one-way ANOVA with Tukey’s multiple comparisons test; p-values: < 0.1234 (ns), < 0.0332 (*), < 0.0021 (**), < 0.0002 (***), < 0.0001 (****). The red line represents mean $\pm$ SD. *AKS* acute kidney slices, *NTS* nephrotoxic serum, *SD* slit diaphragm, *ns* not significant.

Using our protocol, we obtained data on podocyte calcium levels and structural data of the slit diaphragm architecture in single glomeruli. Analysis of the calcium data revealed that animals of both NTS groups showed significantly elevated calcium levels compared to controls. However, there was no significant difference between the low and high dose NTS groups (Fig. 3a, correlative MP + STED). Analysis of the STED data of sequentially imaged glomeruli showed a significant reduction of slit length in both disease groups (high and low dose NTS) compared to control. Additionally, the higher NTS dose (11 µl/g) decreased the SD length further, indicating dose-dependent damage to the podocyte architecture (Fig. 3c). Importantly automatic and manual analysis of SD length failed in both NTS groups in a subset of glomeruli due to the complete loss of a visible SD structure caused by extensive damage (Fig. 3c, correlative MP + STED). This was the case in 13% of measurements of mice treated with low dose NTS and in 42% of measurements with high dose NTS. For analysis of these glomeruli, the SD length was set to zero.

We then compared these results to previously performed experiments, where we independently performed either only calcium imaging or only STED imaging in mice treated with high dose NTS (11 µl/g). As expected, intracellular calcium levels were equally low in control animals and increased comparably after the administration of NTS in both experiments (Fig. 3a, b). However, a comparison of the SD architecture acquired during correlative imaging and STED imaging alone revealed major differences (Fig. 3c, d). In control animals, the measured SD length/area did not differ significantly between both experiments. However, in animals treated with high dose NTS (11 µl/g) the mean SD length/area was significantly reduced in the glomeruli imaged with our correlative imaging approach compared to STED imaging alone (Fig. 3c, d). This was mainly due to the high percentage of glomeruli with complete loss of SD structure.

**Figure 4:**
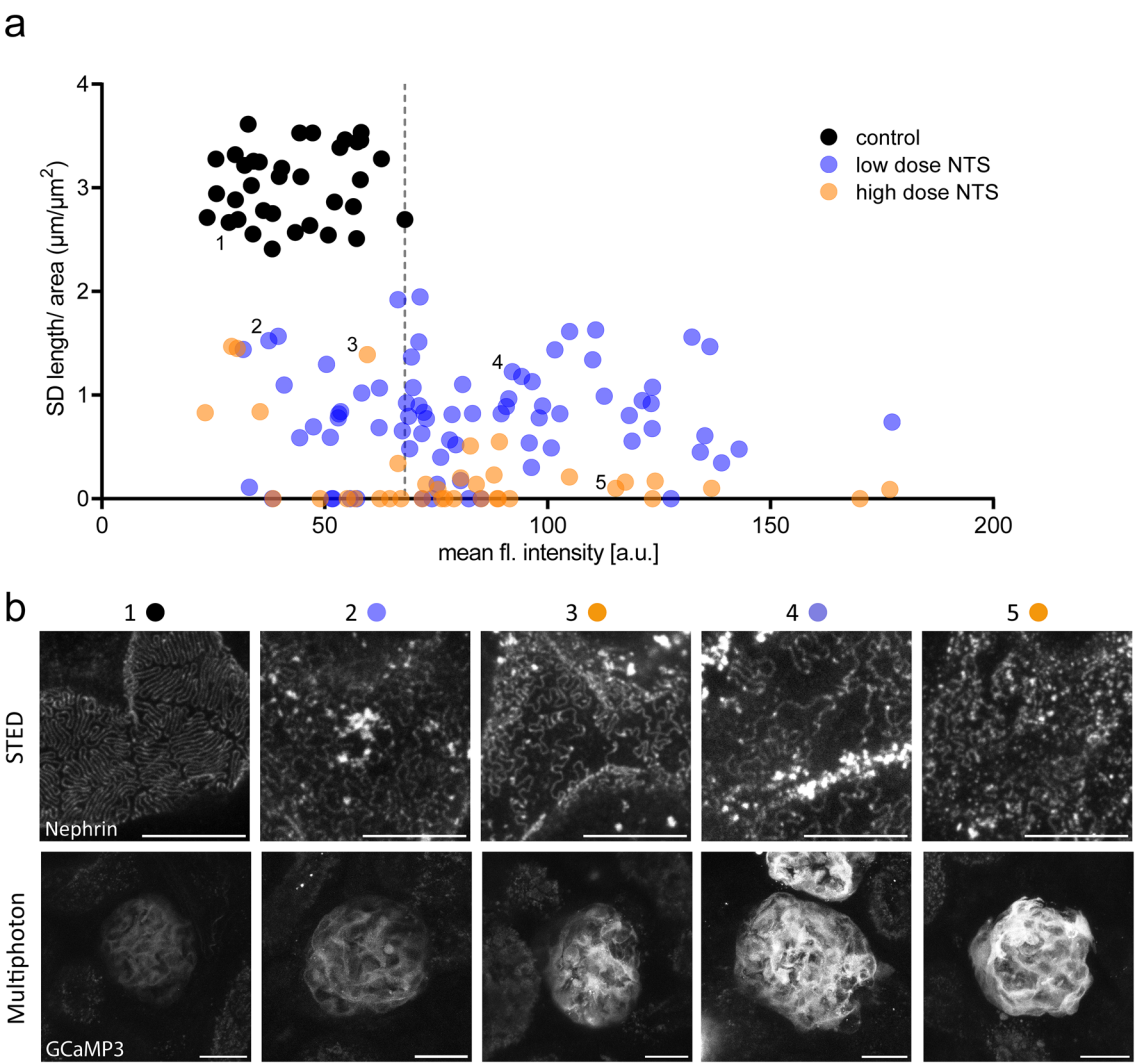
Correlation of intracellular calcium levels and podocyte slit diaphragm ultrastructure. (**a**) Correlation of mean GCaMP3 fluorescence intensities and slit diaphragm length/area for individual glomeruli. The dashed line indicates the maximum GCaMP3 fluorescence intensity measured in control animals. Control: n = 35 glomeruli of 3 mice; low dose NTS: n = 76 glomeruli of 4 mice; high dose NTS: n = 36 glomeruli of 3 mice. (**b**) Representative MIP images of slit architecture acquired using STED imaging (upper panels) and GCaMP3 fluorescence in MP microscopy (lower panels) of the same glomerulus. Symbols above panels show respective positions in panel (a). Scale bar STED – 5 µm; MP images – 25 µm; MIP – maximum intensity projection.

Finally, we investigated a relationship between intracellular calcium levels and SD structure. Therefore, we plotted intracellular calcium levels against SD length/area for each glomerulus measured in our correlative imaging approach. The data did not show a clear correlation (Fig. 4a). In more detail, the plot shows, that 71.1% of glomeruli after low dose NTS and 63.9% of glomeruli after high dose NTS had calcium levels exceeding the maximum calcium levels of controls (indicated by the dashed line in Fig. 4a). While SD damage increased gradually with higher NTS doses, intracellular calcium levels did not. Calcium levels displayed a large variance and showed no significant correlation with the extent of SD damage. Representative images of SD morphology and GCaMP3 fluorescence for selected glomeruli of the different groups are shown in Fig. 4b.

## Discussion

Our novel protocol allows visualization and correlation of functional and ultrastructural information on the level of individual glomeruli. Adding reference points to the AKS tissue by photo ablation during MP imaging enabled the identification of the same glomerulus in STED imaging. It avoided the bias of selecting only glomeruli with residual identifiable SD structure during STED imaging and demonstrated no correlation between intracellular podocyte calcium levels and slit diaphragm architecture. Our data shows, that all glomeruli of control animals exhibited low intracellular calcium levels as previously shown in vivo without spontaneous calcium fluctuations as described in tubular cells^11,24^. To exclude effects on intracellular calcium levels due to the experimental approach we incubated AKS with propidium iodide to label dead cells prior to calcium imaging. Thereby we excluded high calcium levels due to tissue damaged caused by the cutting process or tissue degeneration. Additionally, staining with propidium iodide did not interfere with STED imaging. After induction of NTS nephritis, low and high dose NTS groups showed a similar increase in calcium levels as measured by mean GCaMP3 fluorescence, but with a large variation between glomeruli. In a second set of experiments (Fig. 3c, only MP) the mean fluorescence and the variation of calcium levels were comparable in the control and high dose NTS groups. In addition, NTS had a pronounced dose-dependent effect on SD architecture. Therefore, we are convinced that the NTS model yields reproducible results and the variation in calcium levels is not due to a sampling error of differently affected glomeruli. Nevertheless, we did not find a correlation between increased intracellular calcium levels and the destruction of SD architecture in disease (Fig. 4a). While intracellular calcium levels can change dynamically, morphological changes to the SD take far longer to form and resolve^25^. Therefore, calcium measurements at a single time point could be insufficient to determine the extent of glomerular damage, even if the tight regulation of podocyte calcium levels is lost in disease and mean calcium levels increase. Additionally, the application of a membrane-bound calcium sensor might provide further insights into the calcium dynamics in closer proximity to the SD and thus result in a stronger correlation.

We previously demonstrated that injured podocytes are sensitized to calcium signaling upon stimulation^11^. Therefore it is an intriguing possibility, that not the basal calcium levels, but the susceptibility to calcium signaling itself has a close relationship with the degree of damage. Adding a perfusion pump to exchange buffers to our setup would allow for triggering dynamic calcium responses with compounds such as angiotensin II or ATP and correlate them to podocyte ultrastructure by STED microscopy to test this hypothesis.

Additionally, our correlative imaging approach revealed that in nearly half of the glomeruli in the high dose NTS group nephrin abundance was diminished to such a degree that analysis of SD length by STED imaging was not possible (Fig. 3c). The main difference between correlative imaging and STED imaging alone is the selection of glomeruli during initial calcium imaging. The fact that the mean fluorescence of the calcium indicator GCaMP3 was comparable with a previous set of experiments argues against a selection bias towards highly damaged glomeruli in this step. Glomeruli with a complete disruption of the SD architecture lack the continuous nephrin staining pattern and they are likely to be overlooked during STED imaging alone. By picking intact glomeruli in living tissue during the first imaging step, we eliminated the selection bias based on the recognizability of the slit diaphragm pattern. Thus, we acquired glomeruli with more extensive SD damage than STED microscopy alone would (Fig. 3c, d). Therefore, the published SD analysis protocol presumably underestimates the severity of alterations in podocyte architecture in advanced cases of glomerular disease^15^.

Other groups also reported the use of MP microscopy systems with an implemented STED laser beam for increased resolution, but as no fluorescent reporter of the SD is available, this does not allow visualization of the SD^19,20,26^. In contrast our correlative imaging approach combines vital tissue calcium imaging and high-resolution imaging of antibody labelled proteins within the same sample. Although it requires two imaging sessions, separate MP and STED microscopes are more widespread available than the combination of both lasers in a single microscope.

The increasing availability of genetically encoded fluorescent sensor proteins for in vivo imaging and immunofluorescent labeling options for STED microscopy make our correlative workflow suitable for answering a broad range of research questions far beyond the glomerulus in the kidney. Additionally, by using mosaic expression to obtain only a few positive cells of interest, the resolution could be further improved to correlate microscopy results on the level of individual cells^27^.

In conclusion, we present a protocol for correlative imaging of AKS with MP and STED microscopy to correlate functional and structural information on the level of individual glomeruli.

## Methods

### Mice

Pod:cre-GCaMP3 (C57Bl6/N) mice of both sexes at the age of 4 weeks were used. Control mice (n = 3) received no treatment; for NTS nephritis induction animals received a single i.p. injection of NTS (Probetex, PTX-001-S, San Antonio, TX, USA): low dose 8 µl/g (n = 4) or high dose 11 µl/g (n = 3). No method of randomization or blinding was used. Mice were bred and kept in individually ventilated cages under specific-pathogen free conditions in an in vivo research facility (CECAD, Cologne). Mice were monitored for weight loss and proteinuria starting on day 3 (not shown). After 5 days mice were euthanized by cervical dislocation and the kidneys were harvested. All experiments were approved by the LANUV NRW, Germany, with the authorization number: VSG 81-02.04.2018.A351. Animal experiments complied with LANUV regulations and the ARRIVE guidelines^28^.

### Preparation of acute kidney slices (AKS) and MP microscopy

Freshly harvested mouse kidneys were embedded in low-melting agarose (4%) and cut into 300 µm slices using a vibratome (Leica, VT1200 S). Slices were maintained in Krebs-Henseleit-Buffer carbogenated with 95% O_2_/5% CO_2_ before and during the experiment^29^. To enable identical slice orientation in both imaging modalities, a triangle-shaped piece of tissue was cut from the AKS at the upper left corner. To ensure tissue integrity and exclude glomerular damage caused by cutting, AKS were incubated with propidium iodide (5 µM, Sigma) for 5 min. Glomeruli, which contained cells with nuclear propidium iodide staining, were excluded from the analysis. MP imaging was performed with a TCS SP8 MP-OPO microscope (Leica Microsystems) with a Chameleon Vision II laser (Coherent) tuned to 940 nm within a maximum of 5 h after harvesting the kidneys. Burning marks for reorientation were created by laser ablation using the ‘bleach point’ tool of the Leica Software. Single field of view z-stacks of glomeruli were acquired with 512 × 512 pixel and around 50–60 µm total stack size. After MP imaging, all AKS were fixed with 4% formaldehyde overnight.

### Preparation of acute kidney slices for STED microscopy

Immunofluorescent labeling was conducted as previously described^14^. Shortly, all fixed AKS were optically cleared, followed by consecutive primary and secondary antibody incubation in PBS-T (0.1% Trition-X100 in PBS) for 24 h at 37 °C. If only STED microscopy was performed a piece of the kidney was immersed in hydrogel, the gel was polymerized for 3 h at 37 °C and ultimately washed with PBS. The piece was then cut into 200 µm slices using a vibratome (Leica, VT1200 S). The slices were then processed in an identical way as AKS.

Primary antibodies used: guinea pig anti-nephrin (Fitzgerald, 20R-NP002, 1:100), anti-GFP-Alexa647 (Thermo Fisher, A-31852, 1:100) for labelling GCaMP3. Secondary antibody: donkey anti-guinea pig Atto594 (coupled in house, 1:100). Confocal and STED images of fixed samples were acquired with a TCS SP8 gSTED 3X microscope (Leica Microsystems) equipped with a white light laser and a 775 nm 2D donut STED depletion laser (Leica Microsystems). The excitation wavelengths were: λ_Ex_ = 594 nm for Atto594 and λ_Ex_ = 650 nm for Alex647, channels were acquired sequentially. We obtained images with a resolution of 0.011 µm per pixel, with a 2048 × 2028 pixel image size. For each glomerulus, only one nephrin-stained capillary surface was acquired as close to the coverslip as possible with a z-stack size of approximately 2–4 µm depending on the orientation of the capillary.

### SD length analysis

SD length was measured using a previously published macro^15^. The analysis was conducted as described in detail in the original publication. The macro itself is deposited on GitHub: https://gist.github.com/github-martin/e699d18ae6cfa5b1a6aace15d3c3544c. In a few cases, where the image contrast was insufficient to use the macro, the SD length was measured manually using FIJI.

### Calcium measurements

Calcium levels in podocytes were measured using FIJI and analyzed as previously published^11^. Briefly, maximum intensity projections of z-stacks of glomeruli were manually annotated with an ROI compromising the glomerular area. The mean fluorescence intensity of this ROI was used as a readout.

### Statistics

For statistical analysis of mean fluorescence intensities and SD length per area, we used ordinary one-way ANOVA with Tukey’s multiple comparisons test. No data points were excluded. P values < 0.05 were considered statistically significant.

### Supplementary Information

Supplementary Information 1.

Supplementary Video 1.

## Data Availability

The datasets generated and analyzed during the current study are available in the figshare repository, https://figshare.com/s/79be57e74e3fe693876b with the reserved 10.6084/m9.figshare.24118053.
