# Supplementary material for: Correlative multiphoton-STED microscopy of podocyte calcium levels and slit diaphragm ultrastructure in the renal glomerulus
Source: Sci Rep. 2024 Jun 6;14:13019. doi: 10.1038/s41598-024-63507-9 (PMC11156906; doi:10.1038/s41598-024-63507-9)
Supplement: Supplementary file 1 — Supplementary Information 1. [file 41598_2024_63507_MOESM1_ESM.pdf]

Correlative multiphoton-STED microscopy of podocyte calcium levels and slit diaphragm ultrastructure in the renal glomerulus.

Eva Wiesner[1,2,4], M.Sc., Julia Binz-Lotter[1,2,4], PhD, Agnes Hackl[1,3], MD, PhD, David Unnersjö-Jess[1,2], PhD, Nelli Rutkowski[1,2], M.Sc., Thomas Benzing[1,2], MD, *Matthias J. Hackl[1,2], MD.

[1]Department II of Internal Medicine and Center for Molecular Medicine Cologne, University of Cologne, Faculty of Medicine University Hospital Cologne, Germany.

[2]University of Cologne, Faculty of Medicine and University Hospital Cologne, Cluster of Excellence Cellular Stress Responses in Aging-associated Diseases (CECAD), Germany

[3]Department of Pediatrics, University of Cologne, Faculty of Medicine and University Hospital Cologne, Cologne, Germany

[4]contributed equally

Corresponding author: Matthias J. Hackl, Matthias.hackl@uk-koeln.de, phone: +4922147832319, fax: +492214781422944

Supplementary Information

Supplemental Video 1: Structural overview of the same glomerulus imaged with a multiphoton microscope (left) and confocal settings on a STED microscope after processing (right). The left stack shows a glomerulus of a healthy mouse expressing GCaMP3 in podocytes acquired by multiphoton imaging. The right stack shows the same glomerulus after processing (fixation and staining) acquired with confocal imaging settings using a STED microscope. A comparison of both image stacks confirms the identification of the same glomerulus in both imaging modalities.

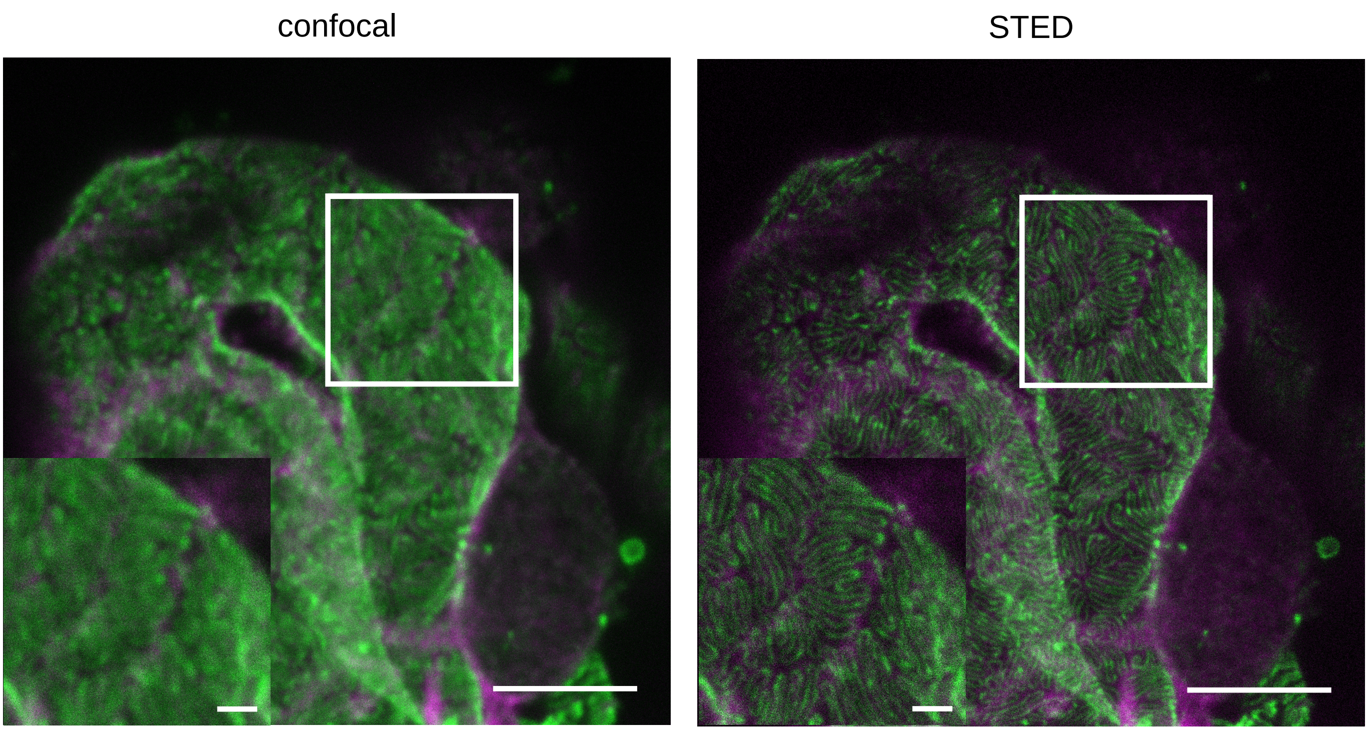

Supplemental Figure 1: Comparison of confocal and STED images of the nephrin-stained slit diaphragm. Left image shows a MIP acquired with confocal settings. Right panel shows the same area imaged with the same settings and additional STED laser beam at 775nm. Green channel shows anti-nephrin Atto594 staining. Magenta channel shows anti-GFP Alexa647 staining. Bottom left corner of images shows zoomed in area (white rectangle).

MIP – maximum intensity projection, scale bars – 5µm; zoom in – 1 µm.
